# Investigation of anti-galectin-8 levels in patients with multiple sclerosis: A consort-clinical study

**DOI:** 10.1097/MD.0000000000032621

**Published:** 2023-01-06

**Authors:** Ufuk Cinkir, Levent Sinan Bir, Selma Tekin, Ahmet Magrur Karagulmez, Esin Avci Cicek, Hande Senol

**Affiliations:** a T.C. Saglik Bakanligi Başakşehir Cam ve Sakura Sehir Hastanesi, Communication, T.C. Saglik Bakanligi Başakşehir Cam ve Sakura Sehir Hastanesi, Istanbul, Turkey; b Pamukkale Universitesi Tip Fakultesi Hastanesi, Communication, Pamukkale Universitesi Tip Fakultesi Hastanesi, Denizli, Turkey.

**Keywords:** B cells, immune system, inflammation, neurodegeneration, T cells

## Abstract

Galectins are a family of endogenous mammalian lectins involved in pathogen recognition, killing, and facilitating the entry of microbial pathogens and parasites into the host. They are the intermediators that decipher glycan-containing information about the host immune cells and microbial structures to modulate signaling events that cause cellular proliferation, chemotaxis, cytokine secretion, and cell-to-cell communication. They have subgroups that take place in different roles in the immune system. The effect of galectin-8 on multiple sclerosis disease (MS) has been studied in the literature, but the results seemed unclear. In this study, we aimed to determine anti-galectin-8 (anti-Gal-8) levels in MS and their potential use as biomarkers.

**Methods:**

In this experimental study, 45 MS patients diagnosed according to McDonald criteria were included in the patient group. The healthy control group contained 45 people without MS diagnosis and any risk factors. Demographic data, height, weight, body mass index, blood glucose, thyroid-stimulating hormone, alanine transaminase, aspartate transaminase, creatinine, low-density lipoprotein, anti-Gal-8 levels, the prevalence of hypertension, diabetes mellitus and coronary artery disease were recorded. In addition, the expanded disability status scale and disease duration were evaluated in the patient group. Data were presented as mean ± standard deviations.

**Results:**

The mean blood anti-galectin-8 value of the patient group was 4.84 ± 4.53 ng/mL, while it was 4.67 ± 3.40 ng/mL in the control group, and the difference in these values was found statistically insignificant (*P* > .05). Moreover, body mass index, glucose, alanine transaminase, aspartate transaminase, thyroid-stimulating hormone, and low-density lipoprotein levels were also statistically insignificant (*P* > .05).

**Conclusion:**

This study examined anti-Gal-8 levels in MS patients. The relationship between MS and galectin-8 and anti-Gal-8 levels in patients needs further clarification. As a result, the study’s results could help elucidate the pathogenesis of MS and give more evidence for diagnosis.

## 1. Introduction

Multiple sclerosis (MS) is a progressive neurodegenerative disease with inflammation, which might present various signs and symptoms. It mainly affects younger females and is typically characterized by firstly demyelination and then axonal degeneration of the central nervous system (CNS).^[1,2]^ MS may result in progressive disability and is more common in Caucasians and females.^[1,2]^ It affects almost 2 million people worldwide.^[1,2]^ Lesions might be observed in white and gray matter, which might cause different symptoms.^[3,4]^ The most common symptoms are loss of sensation, motor strength and vision, dizziness, imbalance, diplopia, and bladder problems.^[3,4]^

Galectins, formerly known as “S-type lectins,” are a subfamily of proteins that bind beta-galactoside carbohydrates with high specificity. They are present in many life forms, from nematodes to animals, performing various functions. Different galectins have been described in humans as responsible for multiple intra and extracellular functions, including tremendous importance in immunity and disease.^[5]^

Galectins (Gals) play critical regulatory roles in the immune system. Its position within MS remains unknown. Recombinant galectin-8 (Gal-8) ameliorates experimental autoimmune encephalomyelitis (EAE). MS is not an autoimmune disease, but since MS is an inflammatory, immunological disease, studying Gals on MS could be helpful to illuminate the relationship between them.^[6]^ We decided to investigate the level of anti-galectin-8 (anti-Gal-8) in MS patients to help to elucidate its potential effects on the CNS.

Studies on anti-Gal-8 levels in MS patients are pretty limited in the literature. Therefore, we aimed to determine anti-Gal-8 levels in MS patients and any potential relationship between its levels with the disease.

### 1.1. Ethical review

Pamukkale University’s noninvasive clinical research ethics committee approved this study.

### 1.2. Participants

MS patients diagnosed according to 2017 McDonald MS criteria (n = 45) were taken into the patient group who had been examined in the department of neurology in the medical faculty of Pamukkale University, Denizli, Turkey. The patients were all in remission. They were not given any steroids. All of the participants were receiving disease-modifying treatments. The time that passed since the last attack was unknown. People without MS diagnosis and any risk factor (MS family history, co-existing certain autoimmune diseases or infections such as systemic lupus erythematosus, Sjogren’s syndrome, Epstein-Barr virus infection, and Lyme’s disease) were included in the control group (n = 45). All members were informed, and an informed consent form was obtained from each participant.

In each group, demographic data, height, weight, body mass index, blood glucose level, thyroid-stimulating hormone, alanine transaminase, aspartate transaminase, creatinine, low-density lipoprotein, triglycerides, and anti-Gal-8 levels were determined, and present hypertension, diabetes mellitus, and coronary artery disease were also recorded. In addition, expanded disability status scale (EDSS) and disease duration were determined in the patient group.

### 1.3. Blood collection

After obtaining informed consent, we took blood samples (5 mL) from the median cubital vein and transferred them into yellow-capped serum tubes containing separating gel. Immediately after blood was centrifuged (2500 × g for 20 minutes at + 40°C), serum was separated and stored in Eppendorf® tubes at -20°C before analyses.

### 1.4. Quantification of anti-galectin-8

Anti-Gal-8 levels were determined by Bioassay Technology Laboratory (Biotech Ltd, Shanghai, China) kits using enzyme-linked immunosorbent assay brought to 25°C on the day of analyses. Absorbance readings were performed at a wavelength of 450nm with a BioTek brand enzyme-linked immunosorbent assay reader. Concentrations were calculated with the Gen 5 program. Within-run coefficient of variation levels for anti-galectin-8 was < 8%, and inter-trial coefficient of variation < 10%. The anti-galectin-8 reading range was between 0.05 and 20.00 ng/mL, while the kit’s sensitivity was 0.03 ng/mL.

### 1.5. Study design

Since the study aimed to determine anti-galectin-8 (anti-Gal-8) levels in MS and their potential use as biomarkers, a randomized controlled cross-sectional clinical research design was employed.

### 1.6. Statistical analysis

Data were analyzed using IBM SPSS 25 (Armonk, New York). Via the power analysis, it was found that if there were at least 90 people in the study, its validity would be achieved with a power of 80% with 95% confidence. Continuous variables were given as mean ± standard deviation, and categorical variables as numbers and percentages. The significance test of the difference between the 2 means was compared to independent group differences when parametric test assumptions were provided. If the parametric test assumptions were not provided, the Mann–Whitney U test was used to compare independent group differences. The differences between the categorical variables were examined by Chi-square analysis. The relationships between continuous variables were analyzed with Spearman or Pearson correlation analyses, and appropriate regression models and the differences between categorical variables were examined with Chi-square analysis. *P* < .05 was considered statistically significant.

## 2. Results

Of the patient group in the study, 31 (68.9%) were female, 14 (31.1%) were male, and the female-to-male ratio was 2.21, which is consistent with the literature (, Supplemental Digital Content 1, Supplemental Digital Content, http://links.lww.com/MD/I296). The mean age distribution of the patient group was 40.0 ± 8.9.

The mean EDSS score was 1.79 ± 1.07, the disease duration was 7.42 ± 5.24, and the attacks’ mean number was 7.7 ± 8.4. The relationship between EDSS, disease duration, and anti-galectin-8 levels was statistically insignificant (*P* > .05).

The mean blood anti-galectin-8 value of the patient group was 4.84 ± 4.53 ng/mL, while it was 4.67 ± 3.4 ng/mL for the control group, and the difference in these values was found statistically insignificant (*P* > .05) (Table 1). All the patients were relapsing-remitting MS.

**Table 1:** The laboratory and physical data of the patient and control group.

Variable	Patient group (n = 45)	Control group (n = 45)	*P*
Age	40.0 ± 8.93	48.33 ± 18.09	.024
Height	166.69 ± 8.95	165.58 ± 8.71	.608
Weight	71.33 ± 13.85	73.33 ± 11.1	.126
BMI	25.77 ± 4.9	26.76 ± 3.67	.452
Glucose	101.82 ± 35.19	110.47 ± 48.04	.099
TSH	2.2 ± 1.67	1.98 ± 1.64	.415
ALT	19.13 ± 11.73	17.82 ± 9.81	.515
AST	17.31 ± 5.96	17.56 ± 5.96	.724
Creatinine	0.72 ± 0.14	0.8 ± 0.18	.015
LDL	111.56 ± 35.25	114.27 ± 31.58	.364
Anti-Galectin-8	4.84 ± 4.53	4.67 ± 3.4	.239

ALT = alanine transaminase, AST = aspartate transaminase, BMI = body mass index, LDL = low-density lipoprotein, TSH = thyroid-stimulating hormone.

## 3. Discussion

This research is one of the few studies investigating anti-galectin-8 levels in MS patients. We investigated whether anti-Gal-8 has a potential role in MS and could be used as a biomarker by determining its blood levels in patients with MS and healthy control groups.

MS is a progressive neurodegenerative disease that primarily affects younger females and is characterized by demyelination and axonal degeneration of the CNS. In addition, MS is one of the leading causes of disability in young and middle-aged people.^[1,2]^ MS may result in progressive disability and is more common in Caucasians and females.^[1,2]^ It is estimated to affect about 2 million people worldwide.^[1,2]^ Lesions might be observed in the white and gray matter, which might cause different symptoms.^[3,4]^ The most common symptoms include loss of sensation, motor strength, vision, dizziness, imbalance, diplopia, and bladder problems such as neurogenic lower urinary tract dysfunction.^[3,4]^

According to certain studies, the MS prevalence in Istanbul was 101.4/1,00,000, while in Edirne, it was 33.9/1,00,000.^[7,8]^ The mean age in Turkey was reported as 41.8 ± 12.0 years, and in the Thrace region of Turkey, it was 40.7 ± 10.6 years.^[7,9]^ The patients’ mean age in our study was 40.0 ± 8.9 years. Worldwide, MS is more common among women. Women with MS are approximately twice as high as men.^[4]^ Of the patient group in our study, 68.9% were female, 31.1% male and the female/male ratio (2.2) was similar to the literature.

In a study of the prevalence of diabetes mellitus, hyperlipidaemia (HPL), and hypertension in MS patients, the rate of HPL in MS patients was not different from the average population.^[10]^ In another study, the rate of HPL in the patient group, was found to be not statistically significant.^[11]^ In our study, the HPL rate in the patient group was also statistically insignificant compared with the control group.

Galectins, a subfamily of proteins that bind beta-galactoside carbohydrates with high specificity, are present in many life forms, from fungi to animals, where they perform various functions. Different galectins have been described in humans as responsible for multiple intra and extracellular functions, including tremendous importance in immunity and disease.^[5]^ They are carbohydrate recognition proteins, and in humans, they are involved in many physiological processes, many of which are directly linked with immunity and disease. Galectins act as actors in the modulation of physiopathological processes. They have been detected in various biological activities, ranging from functional early developmental, vascular processes, cell migration, and regulation of immune system cells to pro-inflammatory and anti-inflammatory resolutions.^[12–14]^ Galectins are involved in pathogen recognition and killing and facilitating the entry of microbial pathogens and parasites into the host. During infection, they are the intermediators who decipher glycan-containing information about the host immune cells and microbial structures to modulate signaling events that cause cellular proliferation, chemotaxis, cytokine secretion, and cell-to-cell communication.^[15–18]^ Gals regulate cell-to-cell communication by acting as soluble cell surface pattern recognition receptors.^[19]^

Gal-8’s role in MS, an autoimmune inflammatory disease of the CNS, remains unknown.^[6]^ Gal-8 is a heterodimer with an N-terminal carbohydrate recognition domain and a C-terminal carbohydrate recognition domain. With its particular heterodimeric structure, Gal-8 is able to mediate cell-cell and cell-matrix interactions. It is expressed in various organs and tissues like synovia, tumors, and osteoarthritis.^[20–27]^ Endothelial cells, which come from lymphatic and vascular vessels, are a great source of secreted Gal-8 in tissues to deliver lectin activity via the surrounding microenvironment and circulating cells.^[25,28–31]^ Some studies show that Gal-8 and other members of this family, like galectin-1, have similar effects on T and B cell responses.^[32,33]^ For example, Galectin-4 is involved in bacterial killing.^[34]^

Gal-8 is found in primary and secondary lymphoid organs.^[35]^ In lymph nodes, Gal-8 messenger ribonucleic acid is located in the paracortical T zone and vascular region.^[30]^ Circulating platelets, dendritic cells (DCs), splenic B lymphocytes, and endothelial cells produce Gal-8 in usual and inflammatory conditions.^[28,33,36,37]^ Recombinant gal-8 (rGal- 8) can induce apoptosis of the immature cluster of difference (CD)4 and CD8 mouse thymocyte subpopulation in vitro via the caspase pathway.^[21]^ This proapoptotic effect of Gal-8 on immature T cells shows its part in the central maturation process and tolerance induction.

On the contrary, in peripheral cells, it is found that rGal-8 has 2 effects on naive CD4 + T cells. If there is no antigen, Gal-8-induced proliferation of naive CD4 + T cells causes an increased expression of IL (interleukin)-2, interferon-γ, and IL-4, the polyclonal expansion of Th1 and Th2 populations and results in inflammatory and autoimmune processes.^[38]^

Proliferative and costimulatory effects of Gal-8 on primary CD4 + T cells are mediated by the CD45 phosphotyrosine phosphatase activity so that it results in the activation of Zeta-chain-associated protein kinase 70 and extracellular-signal-regulated kinase 1/2 pathways.^[35,38]^ Some studies showed that Gal-8 is an extracellular stimulus for the Jurkat T cell line, cell adhesion, and triggering extracellular-signal-regulated kinase 1/2 phosphorylation.^[39,40]^ Gal-8 can induce a substantial proliferation of resting human T cells, but it can also induce cell death or antiproliferative activities.^[38]^ rGal-8 has antiproliferative effects like Gal-8.^[32]^

When the stimulus is limited, 2 roles for Gal-8 on T cells, rGal-8, ameliorate EAE.^[41]^ In another study, it was demonstrated that rGal-8 therapy reduced the severity of experimental autoimmune uveitis pathology by anti-inflammatory effects.^[42]^ In 1 study, Gal-8 knocked out mice with severe EAE, demonstrating Gal-8’s anti-inflammatory effects.^[41]^

B cells are responsible for humoral immunity. They recognize extracellular antigens and then differentiate into antibody-secreting plasma cells. Gal-8 binds to mature B cells associated with plasma cell differentiation and promotes plasma cells. rGal-8 can activate splenic B cell proliferation and induce IL-6 and IL-10 production. Exogenous Gal-8 causes the extraction of immobilized antigens. Also, rGal-8 induces Bruton’s tyrosine kinase and protein kinase B phosphorylation which is a way of activation of B cell receptor signaling.^[30]^ In conclusion, these data indicate that Gal-8 has an excellent role in the cooperation between B and helper T cells.

DCs are the antigen-presenting cells that participate in the production of T cell responses, checking the enormity and the type of the immune response.^[43]^ Gal-8 protein is expressed in monocyte-derived human DCs,^[44]^ and bone-marrow-derived mouse DCs.^[35]^ Extracellular Gal-8 binds to splenic and monocyte-derived DCs.^[36,45]^ rGal-8 activates the secretion of major inflammatory cytokines like IL-6 and tumour necrosis factor- α (tumor necrosis factor-alpha).^[36]^ In addition, Gal-8-activate splenic DCs produce high levels of IL-6 during antigen presentation, which results in costimulatory activity.^[46]^ Gal-8 fully activates DCs. This is presumably one of the mechanisms of stimulating adaptive immune responses. Furthermore, in the inflammatory area, the increase in Gal-8 expression might act as an endogenous mechanism to ignite the innate reaction.^[36]^

Neutrophils are phagocytes enriched with bactericidal properties. They are recruited to an affected area by sequential steps: attachment, rolling, adhesion, and transendothelial migration. It is found that Gal-8 has a vital role in neutrophil function.^[47]^ Gal-8 is a significant modulator of neutrophil migration. It is imprecise if neutrophils express Gal-8 themselves; however, the endothelium might be a source. In the early stages of apoptosis, cells externalize phosphatidylserine (PS). As a rule, it is placed on the inner side of the plasma membrane. In addition to being an apoptotic marker, PS exposure stimulates macrophage-mediated phagocytosis.^[48]^ When there is no apoptosis, some of the Gals family induce PS exposure in neutrophils, resulting in phagocytosis and clearance of living cells.^[49,50]^ This type of cellular turnover is a crucial phase in inflammation resolution.^[51]^ All these data mean Gal-8 has a dual behavior, mainly pro-inflammatory. On the contrary, it is also anti-inflammatory.

In our study, anti-Gal-8 serum levels of MS patients were found to be statistically insignificant compared to the healthy group (*P* > .001). This might support the dual behavior of Gal-8. If Gal-8 had just inflammatory effects, since MS is an inflammatory disease, we would have found statistically lower anti-Gal-8 levels than the healthy group. On the other hand, there were some limitations in our study. Firstly, our patient population had no primary progressive multiple sclerosis or secondary progressive MS. Secondly, our population count was limited (90 participants). Disease subgroups did not evaluate patient and biochemical parameters. Therefore, it is strongly recommended that more comprehensive patient data, including primary progressive multiple sclerosis, secondary progressive MS, and separate subgroups and healthy groups, be further investigated.

In conclusion, Gal-8 has a dual behavior, mainly pro-inflammatory; however, it may have anti-inflammatory functions. The effects of Gal-8 on the immune system need to be studied further.

There is some research about Gal-8 levels and the MS relationship, which remains unclear in the literature. This study is one of a few to assess anti-galectin-8 levels in MS patients and showed a statistically insignificant relationship between serum Gal-8 levels and MS.

We recommend further investigating the role of serum anti-Gal-8 levels in larger groups of MS patients. Serum Gal-8 level could be used as a predictive value for MS patients in the future and provide us with valuable information about the course and pathogenesis of the disease.

## Author contributions

**Conceptualization:** Ufuk Cinkir, Selma Tekin.

**Data curation:** Ufuk Cinkir, Selma Tekin, Ahmet Magrur Karagulmez, Esin Avci Cicek.

**Formal analysis:** Ufuk Cinkir, Levent Sinan Bir, Selma Tekin, Ahmet Magrur Karagulmez, Esin Avci Cicek, Hande Senol.

**Funding acquisition:** Ufuk Cinkir.

**Methodology:** Ufuk Cinkir.

**Project administration:** Ufuk Cinkir, Esin Avci Cicek.

**Resources:** Ufuk Cinkir, Esin Avci Cicek.

**Software:** Hande Senol.

**Supervision:** Ufuk Cinkir, Levent Sinan Bir, Selma Tekin, Hande Senol.

**Validation:** Ufuk Cinkir, Selma Tekin, Hande Senol.

**Visualization:** Ufuk Cinkir, Levent Sinan Bir, Ahmet Magrur Karagulmez.

**Writing – original draft:** Ufuk Cinkir.

**Writing – review & editing:** Ufuk Cinkir, Levent Sinan Bir, Ahmet Magrur Karagulmez, Hande Senol.

## Supplementary Material


