# Supplementary figures and images for: Investigation of anti-galectin-8 levels in patients with multiple sclerosis: A consort-clinical study
Source: Medicine (Baltimore). 2023 Jan 6;102(1):e32621. doi: 10.1097/MD.0000000000032621 (PMC9829274; doi:10.1097/MD.0000000000032621)

## Supplemental Digital Content

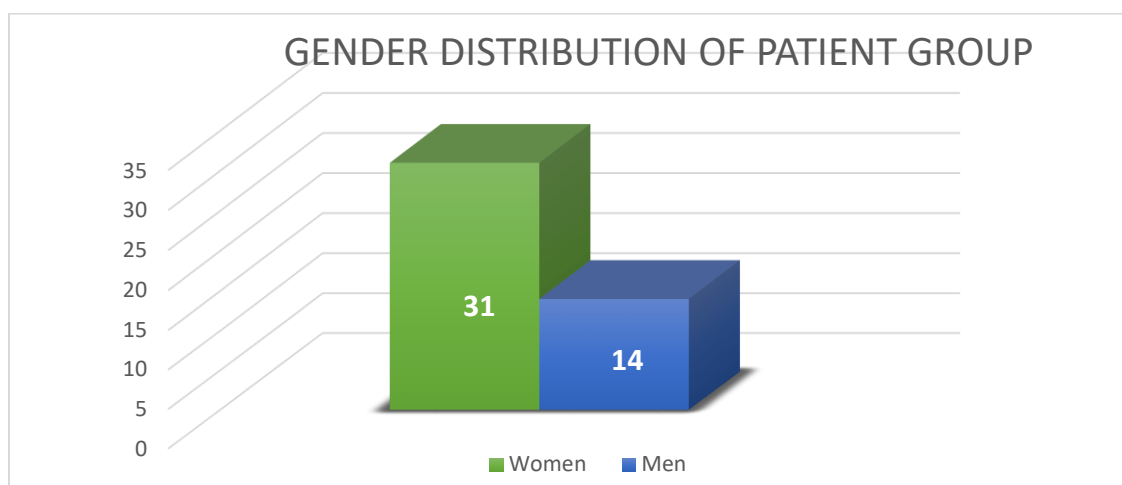

Supplement: Supplementary file 1 [file medi-102-e32621-s001.pdf]
